# Managing Knowledge in Organizations: A Nonaka’s SECI Model Operationalization

**DOI:** 10.3389/fpsyg.2019.02730

**Published:** 2019-12-10

**Authors:** Maria Luisa Farnese, Barbara Barbieri, Antonio Chirumbolo, Gerardo Patriotta

**Affiliations:** ^1^Department of Psychology, Sapienza University of Rome, Rome, Italy; ^2^Department of Political and Social Sciences, University of Cagliari, Cagliari, Italy; ^3^Warwick Business School, University of Warwick, Coventry, United Kingdom

**Keywords:** knowledge management, Nonaka, SECI model, questionnaire, measurement

## Abstract

**Purpose:**

The SECI model (Nonaka, 1994) is the best-known conceptual framework for understanding knowledge generation processes in organizations. To date, however, empirical support for this framework has been overlooked. The present study aims to provide an evidence-based groundwork for the SECI model by testing a multidimensional questionnaire Knowledge Management SECI Processes Questionnaire (KMSP-Q) designed to capture the knowledge conversion modes theorized by Nonaka.

**Methodology:**

In a twofold study, the SECI model was operationalized via the KMSP-Q. Specifically, Study One tested its eight-dimensional structure through exploratory and confirmatory factorial analyses on 372 employees from different sectors. Study Two examined the construct validity and reliability by replicating the KMSP-Q factor structure in knowledge-intensive contexts (on a sample of 466 health-workers), and by investigating the unique impact of each dimension on some organizational outcomes (i.e., performance, innovativeness, collective efficacy).

**Findings:**

The overall findings highlighted that the KMSP-Q is a psychometrically robust questionnaire in terms of both dimensionality and construct validity, the different knowledge generation dimensions being specifically linked to different organizational outcomes.

**Research/Practical Implications:**

The KMSP-Q actualizes and provides empirical consistency to the theory underlying the SECI model. Moreover, it allows for the monitoring of an organization’s capability to manage new knowledge and detect the strengths/weaknesses of KM-related policies and programs.

**Originality/Value:**

This paper proposes a comprehensive measure of knowledge generation in work contexts, highlighting processes that organizations are likely to promote in order to improve their performance through the management of their knowledge resources.

## Introduction

Knowledge represents a crucial drive for organizations’ competitive advantage. It generates value by supporting an organization’s capability to produce innovation (Ahuja and Katila, 2001; Darroch, 2005; Zhou and Li, 2012), learn and unlearn (Hedberg, 1981; Gherardi, 2000; King, 2009), and transfer best practices across boundaries (Hansen, 1999; Carlile, 2004; Patriotta et al., 2013).

Following the wider debate about the emergence of the information age and the knowledge society, recent years have seen an explosion of writings about organizational knowledge from different disciplinary and theoretical perspectives (Patriotta, 2003). The vast literature on knowledge management has documented the processes through which organizations systematically capture, integrate, share, use, and maintain knowledge in order to improve performance. From a managerial perspective, this literature has also suggested the development of management practices able to render knowledge available throughout the organization (e.g., O’Dell and Greyson, 1998; Pfeffer and Sutton, 1999; Brown and Duguid, 2000; Davenport and Prusak, 2000).

Within this plethora of knowledge-based theories, concepts, and tools, the SECI model is widely acknowledged as a theoretical landmark and adopted as framework for most knowledge management conceptualization or descriptive purposes in case studies. The model (Nonaka, 1994; Nonaka and Takeuchi, 1995) considers knowledge creation as a dynamic process, in which the continuous dialog between tacit and explicit knowledge generates new knowledge and amplifies it across different ontological levels (individual, organizational, inter-organizational). The model stands out because it not only formalizes a theory of knowledge creation based on the epistemological distinction between tacit and explicit knowledge but also offers practical tool for assessing knowledge creation in organizational contexts.

Despite the wide diffusion of the SECI model, theoretical development has not always been accompanied by sound methodologies for documenting empirically how the model works in practice (Patriotta, 2004). In particular, the model’s considerable abstraction has led several authors to criticize it, arguing that it is largely based on anecdotal evidence and does not have a sound empirical grounding (Glisby and Holden, 2003; Gourlay, 2006; Bratianu, 2010). Furthermore, owing to its tacit component, the model can be particularly elusive and difficult to test. Indeed, to date only few researches has attempted to operationalize the model, proposing instruments that reflect its four modes (see Nonaka et al., 1994; Becerra-Fernandez and Sabherwal, 2001; Lee and Choi, 2003), but showing inconclusive results.

In order to meet the need for stronger empirical roots in Nonaka’s conceptual model, the present research aimed to propose and validate a new measurement, the Knowledge Management SECI Processes Questionnaire (KMSP-Q). It is conceived of as a multidimensional scale, identifying some key processes related to the four knowledge conversion modes, at different social levels (among individuals, group, and within the organization). This analytic and reliable instrument would allow both to test the SECI model’s consistency and the nature of knowledge generation construct, and to explore its relationship with other variables and make meaningful inferences. For instance, the KMSP-Q provides a systemic picture of the organization’s practices purposing to grow its own knowledge capital, thus enabling to highlight the strengths and weaknesses of specific processes or to verify their effectiveness related to the organizational performance.

This paper starts with a brief description of Nonaka’s SECI model, a conceptual framework for the knowledge creation process. Afterward, we propose an operationalization of this model, depicting organizational processes that concur to generate knowledge, and tested the construct validity of the questionnaire. Specifically, we present two studies: Study One tests the KMSP-Q factor structure on a general sample of employees working in different Italian companies. Study Two examines its generalizability by replicating its factor structure in a sample of employees working in health contexts, which are typical knowledge-intensive sectors. The KMSP-Q criterion validity is also tested by verifying the unique contribution of each knowledge creation process onto different organizational outcomes.

### Theoretical Framework

#### Nonaka’s SECI Model: Types of Knowledge and Their Interaction

Nonaka (1994) conceived knowledge generation as a systemic, dynamic, and ongoing process, which emerges and recurs over time. The SECI root metaphor, the spiral, differs from most knowledge management process conceptualizations, which mainly propose an evolutionary path: for instance, the generation–codification–transfer–application process (Davenport and Prusak, 1998; Ford, 2004); the four processes of creation, retrieval, transfer, and application of knowledge (Alavi and Leidner, 2001); and the accumulation of dynamic competence development (Zollo and Winter, 2002). These models suggest a sequential evolution of knowledge, which has the same quality but a different “stage of life” and usefulness to organizational life, consistent with the commonly accepted conceptualization of knowledge management as a path going from acquiring, storing, and diffusing knowledge to applying it (Chen and Chen, 2006). Conversely, the SECI model focuses on holistic processes that, through knowledge conversion from one type to another, generate a new quality of knowledge.

This conceptualization highlights the underlying processes engendering knowledge, rather than the function that each knowledge stage plays for organizational life. It draws on Polanyi’s (1967) classification regarding the coexistence of two types of knowledge: tacit and explicit, metaphorically comparable to an iceberg. The explicit knowledge represents the part of the iceberg above the water, that is, the knowledge we are aware of and capable of codifying and transferring through formal language. Examples of explicit knowledge in organizations are institutional communications (e.g., newsletters), practices based on formal meetings (e.g., conferences, refresher courses), or knowledge products (e.g., websites, databases, manuals, patents). Explicit knowledge, however, rests on a broad system of tacit knowledge, originated through experience related to professional practice and embedded into the specific work context. This knowledge is situated, analogic, and based on routines and habits (Warnier, 1999). Driving a car or using a computer keyboard are examples of actions based on knowledge we are mainly unaware of. Nonaka and Takeuchi (1995) suggested that knowledge is created through an epistemological process of knowledge conversion from one type to another (tacit and explicit) and amplified through different ontological levels (from interaction between individuals, to groups, to the organization as a whole). The dynamic and continuous interaction between epistemological and ontological dimensions of knowledge gives rise to spiral conversion processes, which quantitatively and qualitatively expand knowledge. It implies that an organization aiming to increase and transform its knowledge should simultaneously promote many and diverse policies and related practices, supporting all of the conversion modes, so that the cycle does not deflate or stop.

The SECI model depicts the four *Socialization–Externalization–Combination–Internalization* conversion modes generated by the switching process from one type of knowledge to another (Nonaka, 1994). The spiral starts with the *Socialization* mode, in which tacit knowledge is exchanged among individuals through shared experiences in day-by-day social interaction. Since tacit knowledge is difficult to formalize and often time- and space-specific, it can only be acquired by directly sharing work experiences (e.g., working side-by-side or observing colleagues). Typically, it is the case of traditional apprenticeship where newcomers learn the tacit knowledge needed in their craft through hands-on routines and close interactions over time (Nonaka and Toyama, 2003). Essentially, this first mode concerns the sharing of tacit knowledge, carried out at an interpersonal level, and allows for the defining of patterns of “how to do things” or reckon events, beliefs, representations of objects, and actions and models of professional practices.

Tacit knowledge is converted, through the *Externalization* mode, into new explicit knowledge in the form of concepts, images, and written documents. Here, individuals use dialog, metaphors, and team confrontations as effective methods to make tacit knowledge codifiable. For this mode to succeed, it is necessary that knowledge is dis-embedded through a reflection-on-action process, inserting distance between the subject and the object (Gherardi, 2000). An important outcome of this reflection on experience is the generation of crystallized knowledge, which is the organizational memory: “members come and go, and leadership changes, but organizations’ memories preserve certain behaviors, mental maps, norms, and values over time” (Hedberg, 1981, p. 6). This formalization leads to new knowledge, accessible in the future and available to other co-workers. This is the gist of “synthesizing,” where new meta-knowledge is generated through selection and connected to the established knowledge system in the organization, which allows for the emergence of new models or mental maps (Nonaka et al., 2006).

Explicit knowledge is then pooled with other intra- or inter-organizational explicit knowledge through the *Combination* mode, being merged, edited, or processed to form more complex and systematic explicit knowledge. The creative use of computerized communication networks and large-scale databases can facilitate this mode of knowledge conversion. For example, using ICT, such as groupware, online databases, intranet, and virtual communities to communicate and share information has been the focus of several previous investigations (Koh and Kim, 2004). These information-sharing processes create higher-order knowledge, such as models, best practices, handbooks, and information systems (Van den Hooff and Van Weenen, 2004) that, in turn, may be disseminated even in the absence of interpersonal relationships.

The SECI spiral concludes with the *Internalization* mode, where explicit knowledge is absorbed by individuals, enriching their tacit knowledge base: formal knowledge is connected to personal experiences to be subsequently transferred and used in practical situations, becoming the base for employees’ renewed routines. For example, in training programs, trainees can enter a new role by reading documents or manuals about their job/company and reflecting upon them; they may also engage in learning-by-doing, simulations, or trial-and-error sessions. Overall, these training activities allow people to integrate new knowledge within their own mental models and enrich their professional know-how, paving the way to new tacit knowledge generation. This new internalized knowledge is re-circulated in the spiral of knowledge, initiating further conversion processes. Conversion modes as a whole and in their interaction give rise to the spiral of knowledge generation (Nonaka, 1994).

#### Some Conceptual and Measurement Issues

**FIGURE 1:**
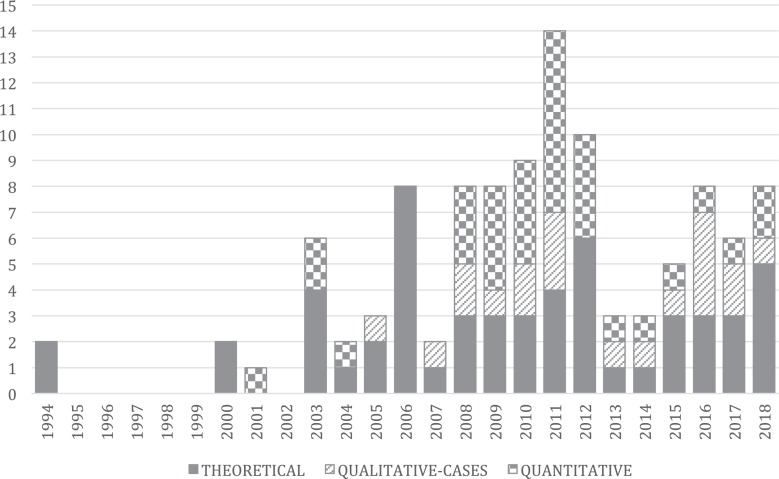
Qualitative review of publications on the SECI model. To identify publications for inclusion in this review, we searched WoS databases using specific keywords linked to the Nonaka’s model, such as “SECI” and “Nonaka,” “application,” “process,” “inventory,” “questionnaire,” or “scale.” We also used a snowball approach by searching the references of relevant publications to identify further papers for inclusion in the review. Inclusion criteria were: (a) publications that were focused on the SECI model; (b) temporary lag from 1994 to December 2018; (c) scholarly publications (conference papers, dissertations, working papers, and practitioner publications were removed). The final sample included 108 publications. References related to this table are available from the correspondence author.

Although SECI model is recognized as the most relevant and comprehensive theoretical proposal in the field of knowledge management and a reference point for many subsequent conceptualizations and studies (Argote et al., 2003), empirical evidence to bolster the model’s consistency is fragmented. Figure 1 depicts results of 108 publications that have focused on the SECI model, published since Nonaka’s (1994) seminal paper on knowledge creation. As shown, the SECI model has mainly been used in theory or for descriptive purposes and case studies; indeed, approximately half of the studies are theoretical articles (*n* = 55), whereas the remaining empirical studies include qualitative case studies (*n* = 20) and quantitative investigations (*n* = 33) with high empirical heterogeneity (Gourlay, 2006).

Furthermore, only some empirical studies have tested the model’s dimensionality, for instance showing that different knowledge processes are associated to specific domains of knowledge (Byosiere and Luethge, 2008) or are related to distinct outcomes (Alavi and Leidner, 2001; Chen and Chen, 2006). Some studies have also found that the effectiveness of a knowledge management process depends on the circumstances under which it was used; thus, individuals may employ different types of knowledge creation processes in order to better perform different types of tasks (Becerra-Fernandez and Sabherwal, 2001; Chou and He, 2004). Nonetheless, these findings are still exploratory, and more research is needed to give empirical support to a multidimensional conceptualization of the knowledge generation process and to demonstrate that it provides more information than a holistic one. More generally, very few studies have verified the SECI predictive validity and tested its effectiveness in generating new knowledge in organizations, thus paving the way to better performance and value creation (see Tammets’, 2012 qualitative meta-analysis, 2012).

Other scholars have questioned the SECI model’s generalizability, highlighting the need to explore its cross-cultural transfer and replication (Glisby and Holden, 2003), the role of contextual factors and external knowledge inputs in shaping knowledge generation (Bereiter, 2002; Martin-de-Castro et al., 2008; Magnier-Watanabe et al., 2011), and the contribution of local social practices (Nonaka and von Krogh, 2009).

A further issue is related to the quality of the new knowledge generated in the conversion processes, that is, whether tacit and explicit knowledge are dichotomic qualities of knowledge rather than poles of a continuum (Nonaka and von Krogh, 2009) or the need to differentiate implicit and tacit knowledge (Li and Gao, 2003). Some scholars have speculated that not all the conversion processes composing the SECI model are truly generative and have called for further research to shed light on this issue (Gourlay, 2006; Bratianu, 2010). Specifically, they have assumed that only those processes that actually change the quality of knowledge (from tacit to explicit or *vice versa*) should be strictly considered knowledge conversion processes, by this generating new knowledge, whereas processes limited to sharing the same quality of knowledge (tacit-to-tacit, explicit-to-explicit) should be more properly conceived of as knowledge transfer processes.

In general, authors have highlighted that Nonaka tended to adopt a philosophical perspective, which made it difficult to actualize the model into a measurement instrument and test its validity (Gourlay, 2006; Tammets, 2012). To date, there are few questionnaires that have attempted to assess the knowledge creation process based on the four SECI conversion modes. The first questionnaire is the one proposed by Nonaka et al. (1994) to study management commitment toward knowledge management practices, assessing the amount of time they spend on specific knowledge creation activities. Subsequently, Becerra-Fernandez and Sabherwal (2001) identified several knowledge management tools examining the presence–absence of each. Also, Lee and Choi (2003) proposed a scale to test a model that linked knowledge management enablers to the SECI modes, and in turn organizational performance. Lastly, Martin-de-Castro et al. (2008) proposed a further questionnaire, but results did not confirm the theoretical structure and were inconsistent between the two independent samples. Despite being conceived as multidimensional questionnaires, they have often been empirically used as unidimensional or not based on validation analyses. Moreover, most of them have targeted managers, thus reducing the possibility of examining employees’ point of view. Overall, inconsistency of results and the absence of a reliable measurement make it difficult, to date, to ascertain the SECI model conceptualization and its criterion validity, for instance, testing its effectiveness in increasing organizational performance (Gourlay, 2006; Tammets, 2012).

#### Aims of the Studies

Drawing from the literature depicted above, to address scholars’ claim for a stronger evidence-based support for the SECI model (Gourlay, 2006), the present study aimed to contribute to the knowledge management literature by generating and validating a multidimensional questionnaire (KMSP-Q) to assess Nonaka’s SECI model. Indeed, a reliable instrument capable of capturing the nature of the construct and providing support for SECI’s multidimensionality would allow organizations to assess whether and how each process specifically contributes to performance, highlight their relative value in comparison to the other processes, and measure their knowledge capital, also in cross-cultural comparisons (Marr et al., 2003).

Specifically, our first goal was to identify prominent dimensions that could be used to operationalize conceptual constructs related to the conversion modes proposed by Nonaka and transform them into concrete knowledge generation organizational processes (Study One). The factor structure of the questionnaire was investigated via both explorative and confirmative factor analyses, together with the internal consistency of all single dimensions. Furthermore, these analyses were replicated in both a general sample (Study One) and in a knowledge-intensive context sample (Study Two), in order to acquire greater generalizability.

The second goal was to verify the KMSP-Q criterion validity by examining whether and how each of the different knowledge processes provided a specific contribution to different organizational performance outcomes (Study Two). Indeed, the multidimensionality of the questionnaire allowed an examination of not only the relationship between each knowledge process and each of the different organizational outcomes considered, but also the simultaneous and unique contribution of each knowledge process compared to the others.

## Study One

### Scale Development and Dimensionality

The first study proposed a new questionnaire, the KMSP-Q (Knowledge Management Processes Questionnaire), that identifies eight processes related to the four modes for knowledge creation and provides a “state of the art” picture of the organization’s capability to implement knowledge. Generative criteria are explained, and the psychometric properties of the scale are assessed. Specifically, we assumed that the knowledge management generation process includes several dimensions, correlated but differentiated among themselves.

#### Item Development

##### General ratio

Nonaka’s conversion modes are too abstract and not applicable in the field; thus, their operationalization was made through identification of the underlying organizational processes. More specifically, the KMSP-Q was based on the following criteria: *(1) Grounded on SECI’s theory*: each dimension was developed while bearing in mind a specific epistemological feature of knowledge creation (the tacit–tacit, tacit–explicit, explicit–explicit, explicit–tacit shifts) and a main ontological level (i.e., individuals, group, organization); *(2) Generalizability of use*: the KMSP-Q was designed for use in most types of organizations and by all levels of employees (not specifically managers); *(3) Roots within a strategic management framework*: items depicted not only the presence–absence of a specific tool or organizational process related to knowledge generation (e.g., the degree of knowledge sharing among teammates) but the “commitment” and the intentionality of the organization in promoting policies aimed at supporting that process and making it useful for enhancing new knowledge (e.g., sharing knowledge to improve work performance) (Darroch, 2005). Indeed, Nonaka highlighted that, although some learning takes place spontaneously or casually in organizational life, the knowledge generation process fully occurs only if the actors are involved in the process, expressing a commitment to learn, and when processes convey management choices (Nonaka et al., 2000): “Organizational knowledge creation, as distinct from individual knowledge creation, takes place when all four modes of knowledge creation are “organizationally” managed to form a continual cycle” (Nonaka et al., 1994, p. 341).

##### Item generation

Our scale development consisted of three steps. In the first phase, a pool of items related to the eight dimensions were generated from a review of the relevant literature, by a research team. The final list of 83 items was subjected to semantic evaluation, and two of the authors assessed their pertinence in respect to the construct. Items were subsequently reduced to 67 and pilot-tested with a sample of employees from different work contexts. Explorative analyses and feedback by participants were discussed and led to the final 48-item and eight-dimension scale (see Supplementary Appendix). The theoretical underpinnings of each dimension are described below. Responses were rated on a frequency scale from 1 (*never or almost never*) to 5 (*very often or always*).

#### Dimensions of the KMSP-Q

The eight dimensions refer to the four SECI conversion processes (two for each process). Among the many possible organizational processes related to knowledge management that could have been included in this model, we chose those that, in our opinion, were more consistent with the epistemological and ontological conversion processes and, at the same time, have been highlighted by the literature as having an impact on organizational effectiveness. In other words, those domains that, if absent within organizational life, would determine the failure of managing their own heritage of knowledge.

The first two dimensions pertain to the tacit-to-tacit conversion mode (*Socialization*), mainly capturing the knowledge exchange at the interpersonal level. *Mentoring practices* relate to tacit knowledge transfer from expert members (supervisors, tenured peers) to newcomers or less experienced members, through tactics designed to support better socialization at work. They allow the sharing of tacit knowledge by observation, modeling, and assimilation of the implicit and unconscious skills embedded in professional practice. Mentoring is a typical organizational socialization tactic to implement employees’ learning, practical abilities, and personal growth in role transitions, enhancing a deep understanding of professional skills, organizational politics and values, as well as leads to several behavioral, attitudinal, and relational outcomes (Becerra-Fernandez and Sabherwal, 2001; Eby et al., 2008; Farnese et al., 2016a). Example item is: *More experienced colleagues provide less experienced colleagues with constructive feedback about their work*.

*Knowledge sharing* refers to the willingness to share one’s own knowledge (e.g., experiences, best practices, skills) with colleagues, when needed or asked. The literature widely acknowledges the importance of knowledge sharing for organizational performance (van Wijk et al., 2008; Wang and Noe, 2010), but also highlights the difficulty of sharing the knowledge embedded in individuals (Szulanski, 2000). Thus, to promote “lateral communication” and access to individuals with relevant knowledge, social and motivational systems and human resource practices need to be implemented (Alavi and Leidner, 2001; Cabrera and Cabrera, 2005). An example item for this dimension is: *Each one’s know-how is made available to colleagues to deal with problems that may arise.*

The third and fourth dimensions refer to the tacit-to-explicit conversion mode (*Externalization*) in which teammates need to engender a shared language that gives meaning to their actions. Indeed, this conversion mode is mainly focused on knowledge exchange at the group level, paving the way to the building of a wealth of common knowledge. *Team reflexivity* expresses the process of collective reflection on the way we work to critically revise goals, methods, practices, and the environment where they operate, accordingly planning changes to be more effective (West et al., 2000) and enhancing organizational performance and innovativeness (Schippers et al., 2015; Farnese and Livi, 2016). According to Nonaka, “organizations continuously create new knowledge by reconstructing existing perspectives, frameworks, or premises on a day-to-day basis” (Nonaka et al., 1994, p. 341). Through dialog and discussion on experience, employees separate themselves from professional practice. Tacit knowledge is extracted and made explicit through processes of abstraction (e.g., maps) or symbolization (e.g., metaphors), generating higher awareness and a meta-level learning (Gherardi, 2000; West et al., 2000). An example item for this dimension is: *At the end of each project, we examine the mistakes made in order to prevent their repetition in the future.*

*Organizational memory* includes the storage, organization, systematization, and retrieval of past experience and events, aimed to decrease forgetting. Through a disembedding process from individuals and from specific contexts, organizational memory reduces knowledge stickiness to individuals (Szulanski, 2000) and allows teammates to select relevant knowledge. At the same time, it makes experience accessible over time and to other colleagues through a crystallization process connecting it to the wider organizational knowledge system (Wexler, 2002; Nonaka et al., 2006). Practices for memory are based on formalization of experience, for instance, collecting good practices or producing manuals, reports, and other written documentation (Alavi and Leidner, 2001). Scholars have shown that stored knowledge may enhance organizational performance, helping to properly act routines, but also innovation, by supporting access to a stock of expertise and core capabilities (Moorman and Miner, 1997). An example item for this dimension is: *Activities are monitored by collecting and processing relevant data*.

The fifth and sixth dimensions concern the explicit-to-explicit conversion mode (*Combination*). This mode mainly captures the knowledge exchange at the organizational level; indeed, it aims to create and support a knowledge system to make information accessible to all organizational members when needed. *Organizational communication* focuses on establishing norms and formal practices (e.g., meetings, internal communication tools) to share information and news, to keep all members updated and to overcome unit boundaries and hierarchical levels. Thus, a systemic view of the organization is enhanced. By managing the “politics of information” and making information available, the organization lays the cultural conditions for fair distribution of knowledge power and trustworthiness among people (Davenport and Prusak, 1998; Ipe, 2003). The literature has widely supported the relationship of organizational information sharing with performance and innovation (Collins and Smith, 2006; Mesmer-Magnus and DeChurch, 2009). An example item for this dimension is: *We are kept informed about what happens within the organization.*

*Technological support* refers to the contribution of knowledge management systems and tools that boost quick and useful transfer and access to knowledge (Alavi and Leidner, 2001). It is a critical dimension for the success of the organization, because it can be used to systematize, improve, and exchange intra- and inter-firm knowledge, enhancing its competitiveness (Melville et al., 2004). It expresses the willingness to use these tools and to encourage collaborative environments based on reciprocity and knowledge sharing, as well as to facilitate the management of information allowing its systematization, categorization, or reconfiguration (Nonaka, 1994; Goh, 2002). An example item for this dimension is: *Technologies allow us to easily share knowledge and information between different units.*

Finally, the seventh and eighth dimensions concern the explicit-to-tacit conversion mode (*Internalization*) that could be defined as the exercise of operational knowledge. This is a process of embodiment of collective and explicit knowledge that, through practice and reflection becomes a “sticky” individual knowledge (Szulanski, 2000). Thus, this conversion mode mainly focuses on the processes rooting the knowledge at the individuals’ level. Indeed, according to Nonaka and Nishiguchi (2001), knowledge is often in the eye of the beholder, and one gives meaning to a concept through the way one uses it. The *human resources training* dimension is related to those learning processes designed to support employees to assimilate new knowledge and mold their maps, for decision-making and work processes or support role transitions (Salas et al., 2012). Training programs strengthen human and social capital, producing effective advantage for the organization and helping it to remain competitive (Arthur et al., 2003; Alvarez et al., 2004). An example item for this dimension is: *Employees’ work skills are developed through training.*

*Human resources development* refers to all those policies and practices able to support the development of human resources and allowing people to make sense of what they do, to attribute meaning to their professional experience, and to value their extra-role behaviors. A learning organization “encourages continuous learning and knowledge creation at all levels […], defines processes for facilitating the circulation of knowledge […] translating this knowledge into changes in internal and external behavior” (Senge, 1990, p. 21). Overall, it expresses the organization’s capability to be a context where all members are encouraged to learn and to develop their full potential, and human resource development is a core strategy (Argote et al., 2003). An example item for this dimension is: *We have time/resources to reflect upon how to improve our work.*

### Materials and Methods

#### Participants and Procedure

Participants of Study One were 372 Italian employees working in different productive sectors (e.g., industry, service companies, ICT, local public administrations). Respondents were balanced for gender (48.4% males, 51.1% females, two missing). Age ranged from 20 to 61 years (*M* = 37 years, *SD* = 8.8). Participants had attained a relatively high level of education (45.0% high school, 41.5% graduates), held different organizational positions (53.8% operatives, 23.5% technical-specialized, 19.9% supervisors, 2.7% management), had mostly a permanent job (67.8%), and ranged in organizational tenure from 1 to 36 years (*M* = 8.4 years, *SD* = 7.8).

Data were collected through anonymous questionnaires, which were administered by hand delivery and returned. Administration was conducted through the snowball technique, which involved bachelor students who voluntarily took part in the data-collecting phase after a training session. To ensure heterogeneity of the sample, each research assistant approached between 10 and 30 employees from different organizations. Participants voluntarily participated in the study and did not receive any kind of reward. All participants were informed of the anonymity and confidentiality of the survey. The study that collected the data for this validation was approved by the Comitato I.R.B. (Institutional Review Board), Department of Psychology, Sapienza University of Rome (Prot. No. 0000151).

#### Data Analyses

In order to assess the dimensionality of the KMSP-Q, an Exploratory Factor Analysis (EFA) was conducted on the final 48 items. From a theoretical point of view, an eight-factor solution was expected. Afterward, the emerged factor solution was further tested via Confirmative Factor Analysis (CFA) by testing two nested models: Model 1, where all dimensions collapse into a general knowledge management factor; and Model 2, where each of the eight dimensions represent correlated but differentiated factors.

Exploratory Factor Analysis was performed with SPSS 25, while CFA was run with LISREL 9.2.

### Results

#### Exploratory Factor Analysis

In order to examine the dimensionality of the KMSP-Q, an EFA was conducted on the 48 items with Principal Axis Factoring extraction. An oblimin rotation was then performed to reach a simple solution. Both the scree plot (Cattell, 1966) and parallel analysis, based on Monte Carlo simulations (Horn, 1965; Patil et al., 2008), suggested that eight factors should be retained, accounting for 70.9% of the total variance. All of the items of each dimension loaded onto the same factor with a loading greater than 0.35, showing no significant cross-loadings with other factors (see Table 1).

**TABLE 1:** Factor loadings of the explorative factor analysis (PAF with oblimin rotation).

**Items/Factors**	**1**	**2**	**3**	**4**	**5**	**6**	**7**	**8**
DEV_4	0.73							
DEV_3	0.65							
DEV_6	0.53							
DEV_9	0.52							
DEV_8	0.50							
DEV_5	0.43							
TECH_4		0.83						
TECH_5		0.80						
TECH_3		0.76						
TECH_2		0.73						
TECH_7		0.73						
TECH_1		0.59						
SHA_3			0.82					
SHA_5			0.74					
SHA_7			0.72					
SHA_4			0.71					
SHA_2			0.70					
SHA_6			0.47					
MENT_4				0.85				
MENT_3				0.74				
MENT_1				0.62				
MENT_5				0.55				
MENT_2				0.54				
MENT_8				0.45				
REFL_2					−0.68			
REFL_8					−0.62			
REFL_1					−0.60			
REFL_3					−0.56			
REFL_9					−0.55			
REFL_4					−0.47			
MEM_4						0.69		
MEM_5						0.69		
MEM_2						0.65		
MEM_3						0.43		
MEM_8						0.38		
MEM_7						0.36		
OCOM_5							−0.73	
OCOM_6							−0.73	
OCOM_4							−0.72	
OCOM_1							−0.65	
OCOM_2							−0.64	
OCOM_8							−0.40	
TRAI_4								−0.70
TRAI_3								−0.61
TRAI_1								−0.59
TRAI_6								−0.52
TRAI_2								−0.51
TRAI_5								−0.43

*DEV, human resources development; TECH, technological support; SHA, knowledge sharing; MENT, mentoring practices; REFL, team reflexivity; MEM, organizational memory; OCOM, organizational communication; TRAI, human resources training.*

The first factor accounted for 43.7% of the variance and referred to the dimension of *HR development* (DEV). Factor loadings ranged from 0.43 to 0.73 (average loading 0.56). The second dimension tapped the *technological support* dimension (TECH) and explained 6.4% of the variance, with factor loadings ranging from 0.59 to 0.83 (average loading 0.74). The third factor represented the *knowledge sharing* dimension (SHA), which accounted for 4.8% of the variance (factor loadings ranged from 0.47 to 0.82; average loading, 0.69). The fourth factor accounted for 4.7% of the variance and referred to the dimension of *mentoring practices* (MENT). Factor loadings varied from 0.45 to 0.85 (average loading of 0.63). The fifth factor reflected the *team reflexivity* (REFL) dimension (3.3% of the explained variance, factor loadings ranged from 0.47 to 0.68, average loading 0.58), while the sixth factor was loaded by items referring to *organizational memory* (MEM) dimension (2.8% of the explained variance, factor loadings ranged from 0.36 to 0.69, average loading 0.53). The seventh factor referred to the dimension of *organizational communication* (OCOM) and accounted for 2.6% of the variance: its factor loadings ranged from 0.40 to 0.73 (average loading 0.64). Finally, the eighth factor regarded *HR training* (TRAI), explaining 2.4% of the variance, with factor loadings ranging from 0.43 to 0.70 (average loading, 0.56). The eight factors were correlated to each other, with a range varying from modest (0.24) to moderate (0.53).

#### Reliability, Descriptive Statistics, and Correlations

The reliability of each dimension was tested using Cronbach α: all dimensions showed very good internal consistency ranging from 0.88 to 0.92. Descriptive statistics and reliability are reported in Table 2, while intercorrelations among dimensions are reported in Table 3. The correlations among variables were substantial. However, their magnitude suggested a good discriminant validity between dimensions. A further test of their distinctiveness, however, was performed conducting a CFA (see next paragraph).

**TABLE 2:** Means, standard deviations, and reliability of each dimension (Study One).

	***M***	***SD***	**α**	**No. of Items**
Mentoring practices	3.80	0.86	0.91	6
Knowledge sharing	3.56	0.72	0.92	6
Team reflexivity	3.46	0.75	0.88	6
Organizational memory	3.23	0.91	0.89	6
Organizational communication	2.97	0.82	0.90	6
Technological support	3.25	0.80	0.91	6
Human resources training	3.03	0.90	0.90	6
Human resources development	2.71	0.81	0.90	6

**TABLE 3:** Intercorrelations among dimensions (Study One).

	**1**	**2**	**3**	**4**	**5**	**6**	**7**	**8**
(1) Mentoring practices	1							
(2) Knowledge sharing	0.63^∗∗^	1						
(3) Team reflexivity	0.62^∗∗^	0.60^∗∗^	1					
(4) Organizational memory	0.58^∗∗^	0.49^∗∗^	0.67^∗∗^	1				
(5) Organizational communication	0.59^∗∗^	0.61^∗∗^	0.65^∗∗^	0.55^∗∗^	1			
(6) Technological support	0.41^∗∗^	0.52^∗∗^	0.57^∗∗^	0.63^∗∗^	0.51^∗∗^	1		
(7) Human resources training	0.66^∗∗^	0.54^∗∗^	0.63^∗∗^	0.65^∗∗^	0.61^∗∗^	0.53^∗∗^	1	
(8) Human resources development	0.59^∗∗^	0.60^∗∗^	0.64^∗∗^	0.55^∗∗^	0.73^∗∗^	0.52^∗∗^	0.68^∗∗^	1

*^∗∗^*p* < 0.01.*

#### Confirmative Factor Analysis

A CFA was conducted in order to test whether the eight-factor solution was optimal as well as examine whether the eight dimensions were sufficiently distinct from each other. Two alternative nested factor models were contrasted and formally compared. In the first model (M1), the fit of a one-factor solution was tested. In case the eight dimensions were not sufficiently distinct to each other, model M1 would demonstrate a satisfactory fit. In the second model (M2), the fit of an eight correlated factor solution was tested. The two models were then compared (M1 vs. M2) to decide which had the best fit. Model fit was evaluated according to the following indices: the Comparative Fit Index (CFI), the Non-Normed Fit Index (NNFI), and the Root Mean Squared Error of Approximation (RMSEA). In particular, CFI and NNFI values between 0.90 and 0.95 are considered acceptable while values over 0.95 are considered very good; on the other hand, RMSEA values smaller than or equal to 0.08 indicate a good fit. Specifically, it was expected that M2 would show a better fit than M1. In this perspective, also the chi-square difference test (Δχ^2^) between the two nested models was performed (Satorra and Bentler, 2001): if the eight factors were sufficiently distinct, a significant decrease in chi-square from M1 to M2 was expected.

Results of the CFA indicated that the one-factor model (M1) did not show a satisfactory fit, *CFI* = 0.88, *NNFI* = 0.87, *RMSEA* = 0.20. Conversely, the eight-factor model (M2) showed a very satisfactory fit, *CFI* = 0.99, *NNFI* = 0.98, *RMSEA* = 0.08. The chi square difference between M1 and M2 showed that there was indeed a significant increase of fit in M2, Δχ^2^_M__1_–_M__2_ = 1499.16, *p* < 0.000. Thus, the eight-factor model (M2) has to be preferred to the one-factor model (M1) (see Table 4).

**TABLE 4:** Confirmative factor analysis: comparison of two nested models (Study One).

**Models**	**CFA**
M1: Single factor	χ^2^_(104)_ = 1755.96, *p* < 0.000
	*RMSEA* = 0.20
	*CFI* = 0.88
	*NNFI* = 0.87
M2: Eight correlated factors	χ^2^_(76)_ = 256.80, *p* < 0.000
	*RMSEA* = 0.078
	*CFI* = 0.99
	*NNFI* = 0.98
	Δχ^2^_M1_–_M2(28)_ = 1499.16, *p* < 0.000

### Discussion

Study One purposed to generate a new questionnaire, the KMSP-Q, grounded on the theoretical framework of the SECI’s model and designed to capture the four knowledge conversion modes that Nonaka supposed could enhance the organizational knowledge assets. Results confirmed that KMSP-Q had good psychometric properties, as well as each of its eight dimensions. Thus, a first contribution of this study is that findings showed adequate robustness of the KMSP-Q, a measurement that could help to actualize and test the theory underlying the SECI. Indeed, this allows scholars to root knowledge management research on a measure that reflects a strong conceptualization, both assessing the knowledge generation construct and integrating it within possible explicative models with other variables. Also, policies and interventions to support knowledge generation and development may be consistently targeted to implement the effectiveness of organizational practices or hinder the generation process.

Secondly, results of this study showed that the eight-factor model had a better fit than the alternative one-dimension model. This means that each dimension is related to the others, but also offers a unique contribution to the construct. In other words, they do not completely overlap and merging all the different facets in a single dimension could be misleading. Overall, findings supported a multidimensional conceptualization of the knowledge generation process, showing as the eight identified key processes provide more information than a holistic measurement. Hence, our tool is consistent with the theoretical model it was drawn on, reflecting in its multidimensional structure a main feature of the Nonaka’s SECI model, and providing initial evidences for a multifaceted conceptualization of the knowledge generation process. Study Two purposes to strengthen this result, both contributing to its generalization and testing whether and how the eight dimensions could outline specific patterns differently contributing to the organizational effectiveness.

## Study Two

### Generalizability in Knowledge Contexts and Construct Validity

The second study firstly proposed to confirm results from Study One, thus contributing to demonstrating the generalizability of the KMSP-Q. Specifically, it replicated the CFA analyses in an independent sample of employees working in knowledge-intensive contexts. This is the case of health organizations, whose effectiveness relies on an intellectually skilled workforce (Alvesson, 2000). Specifically, health organizations show a high level of heterogeneity in the different activities carried out and within working groups and professions (Perrow, 1965); have to solve increasingly complex health problems and handle demands from clinical care activities (Sorrells-Jones and Weaver, 1999; von Nordenflycht, 2010); and base their practices on high-quality knowledge embedded in equipment, products, and organizational routines (Starbuck, 1992; Candy, 2010). It is therefore fundamental for health organizations to manage their knowledge resources, which constitute their key capital.

Study Two further aimed to assess the KMSP-Q’s criterion validity by verifying whether and how the eight dimensions uniquely contribute to different organizational outcomes (i.e., performance, innovativeness, collective effectiveness). Indeed, as previously discussed, the existing literature has suggested that the different conversion modes could lead to different outcomes (Alavi and Leidner, 2001; Becerra-Fernandez and Sabherwal, 2001; Chou and He, 2004; Chen and Chen, 2006). Specifically, taking into account *epistemological feature*, scholars (Gourlay, 2006; Byosiere and Luethge, 2008; Bratianu, 2010) have claimed that conversion modes that substantially transform the quality of knowledge (i.e., Externalization: tacit-to-explicit; Internalization: explicit-to-tacit) are more strictly related to innovation outcomes rather than conversion modes, that are limited to transferring the same type of knowledge (i.e., Socialization: tacit-to-tacit; Combination: explicit-to-explicit). Consistently, we hypothesized that the Externalization (REFL, MEM) and Internalization dimensions (TRAI, DEV) will be related to organizational innovativeness. Considering the *ontological feature*, we further hypothesized that those dimensions related to the interpersonal (Socialization: MENT, SHA) and team levels (Externalization: REFL, MEM) will be more strictly related to group collective efficacy, whereas the organizational-level dimensions (Combination: OCOM, TECH) will be more strictly related to organizational outcomes (i.e., performance and innovativeness).

### Materials and Methods

#### Participants and Procedure

Participants of Study Two included 466 employees working in the health sector (specifically, in 11 Italian Hospitals). They were mainly females (60.4%) and ranged in age from 23 to 69 years (*M* = 42.7 years, *SD* = 9.6). Consistent with their profession, participants had attained a relatively high level of education (14.4% high school, 33.8% graduates, 15.5% Bachelor’s degree, 26.8% Postgraduate degree). They held different organizational positions (53.8% auxiliary nurses, 69.8% nurses, 6.7% head nurses, 20.9% physicians), had mostly a permanent job (73.2%), and ranged in organizational tenure from 1 to 38 years (*M* = 10.7 years, *SD* = 8.8).

Data collection was conducted by research assistants that directly contacted the hospital’s managers and, after their approval, administered the questionnaire. Participants voluntarily participated in the study and did not receive any kind of reward. Each of the employees received the questionnaire in a blank envelope along with a presentation letter, which contained a brief description of the research and its main objectives. Prior to administering the survey, all participants were informed of the anonymity and confidentiality of the survey and were allowed to decline participation if they so choose. The study that collected the data for this validation was approved by the Comitato I.R.B. (Institutional Review Board), Department of Psychology, Sapienza University of Rome (Prot. No. 0000151).

#### Measures

To measure the knowledge management processes, we used the KMSP-Q, as in Study One. As outcome variables, we used two indicators of organizational performance and innovativeness, respectively, aimed to capture employees’ perception about how well their hospital is doing with regard to its own goals (one item: “*To what degree, in the last year, has your hospital*…*achieved a good performance goal?*”) and the degree their hospital is capable of being innovative (one item: “*To what degree, in the last year, has your hospital*…*been innovative?*”) (Van Dyck et al., 2005). Both indicators were rated on a Likert scale ranging from 1 = *not at all* to 10 = *completely*. The third outcome we considered was group collective efficacy (Borgogni et al., 2009), a six-item scale that captures, from the group’s perspective, the shared belief about how well the work group is conjointly capable to organize and relate to each other in order to produce given attainments. We used the hospital ward – that is the clinical unit defined according to clinical diseases (e.g., orthopedics, surgery) – as the referent for measuring collective efficacy. Example items include: “*I believe that my ward is able to*… *fulfill deadlines even in situations of work overload*”; “… *to keep harmony even when tension is high*.” Response choices ranged from 1 = *strongly disagree* to 5 = *strongly agree*.

### Results

#### Confirmative Factor Analysis

A CFA was performed on an independent sample to test the robustness of the factor structure that emerged in Study One. The same data analysis strategy as in Study One was employed. Results of the CFA indicated that the one-factor model (M1) did not show a satisfactory fit (*CFI* = 0.88, *NNFI* = 0.87, *RMSE* = *0.20*, χ^2^_(__104__)_ = 1646.63, *p* < 0.000). Conversely, the eight-factor solution model (M2) showed a very satisfactory fit (*CFI* = 0.99, *NNFI* = 0.98, *RMSEA* = *0.08*, χ^2^_(__76__)_ = 197.95, *p* < 0.000). The chi-square difference between M1 and M2 showed that there was a significant increase of fit in M2 (Δχ^2^_M1_–_M2 (28)_ = 1448.68, *p* < 0.000). Thus, as in Study One, results of the CFA suggested that the eight-factor solution has to be preferred to the alternative one-factor model.

#### Multivariate Regression Analysis

A multivariate regression analysis with manifest variables was conducted in order to test the criterion validity of the KMSP-Q. In this analysis, the eight KMSP-Q dimensions were the predictors while organizational performance, organizational innovativeness, and group collective efficacy comprised the three criteria. Standardized structural coefficients are reported in Tables 5, 6). The three outcomes were predicted by different knowledge management dimensions. Organizational performance was predicted by DEV, REFL, and TECH, and marginally by OCOM. Organizational innovativeness was predicted by OCOM, TECH, and DEV. Group collective efficacy was predicted by REFL, SHA, DEV, MEM, and MENT. The KMSP-Q dimensions accounted from 31 to 40% of the variance of the three outcomes.

**TABLE 5:** Means, standard deviations, and reliability (in parentheses) of each dimension, and intercorrelations among dimensions and outcome variables (Study Two).

	***M***	***SD***	**1**	**2**	**3**	**4**	**5**	**6**	**7**	**8**	**9**	**10**	**11**
(1) Mentoring practices	3.44	0.88	(0.86)										
(2) Knowledge sharing	3.22	0.87	0.54^∗∗^	(0.93)									
(3) Team reflexivity	3.26	0.85	0.57^∗∗^	0.65^∗∗^	(0.88)								
(4) Organizational Memory	2.76	0.96	0.49^∗∗^	0.56^∗∗^	0.73^∗∗^	(0.88)							
(5) Organizational communication	2.85	0.90	0.47^∗∗^	0.69^∗∗^	0.67^∗∗^	0.66^∗∗^	(0.91)						
(6) Technological support	2.86	0.96	0.37^∗∗^	0.61^∗∗^	0.59^∗∗^	0.64^∗∗^	0.67^∗∗^	(0.92)					
(7) Human resources training	2.77	0.95	0.53^∗∗^	0.61^∗∗^	0.69^∗∗^	0.71^∗∗^	0.71^∗∗^	0.67^∗∗^	(0.91)				
(8) Human resources development	2.48	0.90	0.47^∗∗^	0.63^∗∗^	0.63^∗∗^	0.63^∗∗^	0.74^∗∗^	0.62^∗∗^	0.76^∗∗^	(0.91)			
(9) Organizational performance	6.16	2.22	0.31^∗∗^	0.40^∗∗^	0.46^∗∗^	0.45^∗∗^	0.49^∗∗^	0.43^∗∗^	0.43^∗∗^	0.45^∗∗^	–		
(10) Organizational innovativeness	5.60	2.47	0.33^∗∗^	0.54^∗∗^	0.48^∗∗^	0.51^∗∗^	0.57^∗∗^	0.52^∗∗^	0.54^∗∗^	0.53^∗∗^	0.74^∗∗^	–	
(11) Group collective efficacy	3.21	0.82	0.44^∗∗^	0.55^∗∗^	0.55^∗∗^	0.41^∗∗^	0.48^∗∗^	0.41^∗∗^	0.48^∗∗^	0.51^∗∗^	0.41^∗∗^	0.41^∗∗^	(0.92)

*^∗∗^*p* < 0.01.*

**TABLE 6:** Results of multivariate regression analysis.

	**Organizational performance**		**Organizational innovativeness**		**Group Collective efficacy**	
	**β**	***p***	**β**	***p***	**β**	***p***
(1) Mentoring practices	0.037	n.s.	0.002	n.s.	0.107	0.025
(2) Knowledge sharing	0.040	n.s.	0.042	n.s.	0.247	0.000
(3) Team reflexivity	0.174	0.009	0.025	n.s.	0.269	0.000
(4) Organizational memory	0.027	n.s.	0.096	n.s.	0.128	0.038
(5) Organizational communication	0.136	0.050	0.259	0.000	0.004	n.s.
(6) Technological support	0.148	0.014	0.141	0.012	0.007	n.s.
(7) Human resources training	0.062	n.s.	0.111	n.s.	0.025	n.s.
(8) Human resources development	0.197	0.004	0.127	0.047	0.197	0.002
	*R*^2^ = 0.308		*R*^2^ = 0.395		*R*^2^ = 0.398	

β *are standardized regression coefficients.*

### Discussion

Results of Study Two provided good psychometric support for the KMSP-Q’s validity, being consistent with findings from Study One and thus contributing to its generalizability. Specifically, the CFA comparison between the one-factor and eight-factor models supported a multidimensional conceptualization of the construct in knowledge-intensive contexts.

Second, Study Two contributed to further test the criterion validity verifying whether and how the eight KMSP dimensions were related to the outcome variables. The multivariate regressions showed different and specific relationships with the three outcomes, thus providing a unique contribution in explaining processes that, from knowledge generation, lead to better overall organizational effectiveness. Nevertheless, future longitudinal studies should confirm the predictive validity of the eight dimensions. Specifically considering our hypotheses, results were consistent with the ontological feature of the SECI model, but not with the epistemological one. Indeed, considering the *ontological feature*, all of the interpersonal-level (MENT, SHA) and team-level (REFL, MEM) dimensions were significantly related to group collective efficacy, as expected. Also, the two organizational-level (OCOM, TECH) dimensions were related to organizational performance and innovativeness indicators. The two individual-level dimensions showed specific patterns: TRAI did not provide any unique contribution to the considered outcomes while DEV significantly contributed to all of them. These findings suggest that while training processes support individual knowledge creation or implementation, its actual transfer into organizational practices and subsequent outcomes depends on the training design and other organizational conditions (Arthur et al., 2003; Salas et al., 2012).

Taking into account the *epistemological level* and following scholars’ suggestions (Gourlay, 2006; Byosiere and Luethge, 2008; Bratianu, 2010), we hypothesized that dimensions related to the transformative modes (Externalization: REFL, MEM; Internalization: TRAI, DEV) may be more strictly related to innovation. Results demonstrated that only DEV was related to organizational innovativeness while REFL was related to organizational performance but not to innovativeness. We propose that these positive or missing links may be due to our innovativeness indicator that measured the actual implementation of innovation rather than the creative preliminary stages for innovativeness. Thus, it is plausible that all the four conversion models contribute to innovation, but at different phases of the process (i.e., idea exploration, generation, championing, and implementation). For instance, some scholars have highlighted that reflexivity practices set the conditions for readiness to innovation but do not necessarily result in its implementation (West, 2000; Farnese and Livi, 2016; Farnese et al., 2016b). Conversely, both performance and innovation indicators were related to the two macro-level knowledge-transfer dimensions (OCOM, TECH), expressing the more explicit and formalized quality of knowledge. Future studies should verify whether dimensions expressing different conversion modes differently relate to creativity and implementation of innovation stages (Lee and Choi, 2003) or also how the interplay among them paves the way to innovation through different patterns.

## General Discussion

Scholars have recognized the prominent role of the SECI model in offering a comprehensive conceptualization for organizational knowledge generation processes. However, extant research is grounded on limited empirical evidence with respect to both the model’s operationalization and its contribution to organizational effectiveness (see our qualitative review and Tammets’, 2012 meta-analysis, 2012). To address this issue, the aim of this research was to develop and test a multidimensional instrument, the KMP-Q, to capture the generation modes leading to knowledge creation. Our results provide initial empirical evidence for the KMSP-Q’s structure, showing its psychometric robustness and supporting the usefulness of a multidimensional conceptualization.

Overall, the twofold studies contribute to the knowledge management literature in several ways. Firstly, we propose an overarching measure of knowledge generation processes in work contexts (Alavi and Leidner, 2001), consistent with the epistemological and ontological SECI features. This is an important starting point because a reliable instrument allows for the proposal of explicative models or the testing of specific patterns that lead to different indicators of organizational effectiveness (Becerra-Fernandez and Sabherwal, 2001; Marr et al., 2003). Thus, having a reliable instrument is the essential premise for testing the SECI model. Future research could assess its consistency and generalizability based on a sound empirical ground, also in cross-national or cross-sector studies (Glisby and Holden, 2003; Gourlay, 2006; Bratianu, 2010); verify its validity in predicting value creation (Tammets, 2012); or focus on organizational factors that affect the implementation of knowledge management strategies (e.g., goals and values consistent with learning-oriented practices).

Secondly, in line with Nonaka’s framework, results of both studies gave support to a multidimensional conceptualization of the knowledge generation process, showing that the different key processes hold a unique informative contribution that add to the overall organization’s capability to generate knowledge, thus leading to different outcomes.

A further finding is related to the prominent role of the ontological feature that, highlighting the social level where knowledge is generated, paves the way to specific outcomes. In other words, a multidimensional operationalization that distinguishes among processes at the interpersonal, group, and organizational levels allows us to focus on how each social level contributes to generate new knowledge. This represents a starting point for future research to analyze how each social level may enhance or hinder the flow of knowledge production. For instance, it can explore whether and how communication exchange works among teammates (i.e., at group level) and through top-down levels or how to implement knowledge retention (e.g., memory, forgetting) at the individual and organizational level.

Conversely, the epistemological feature didn’t seem to discriminate among transformative or generative modes, in relation to the considered outcomes. This result could suggest that, consistently with Nonaka’s model, all modes fairly contribute to the spiral of new knowledge. Following other scholars’ suggestions (see Gourlay, 2006; Bratianu, 2010), further research should verify this result, for instance by relating the KMP-Q dimensions to different innovation outcomes.

Finally, this study offers a cross-cultural contribution to the model given that, as far as we know, research on SECI in western countries is underrepresented, probably due to the less promising results emerging from several studies in Western organizations, which have shown how much national cultures can significantly promote or hinder the success of knowledge management initiatives (Tseng, 2010; Magnier-Watanabe et al., 2011).

### Practical Implications

The KMP-Q represents a useful inventory for management in order to monitor their capability to manage knowledge resources, feature their profile with relative strengths and weaknesses, and assess organizational investments on policies and practices knowledge oriented.

Specifically, the KMSP-Q provides management with more insight into the “state of the art” of organization’s capability to generate and implement its own knowledge, tapping critical processes. Assessing a profile is important because the SECI model assumes that knowledge creation is an endless and recursive process. Thus, criticalities in a knowledge conversion mode (epistemological feature) or on a social level (ontological feature) could create an impasse or even a break in the flow of knowledge.

Moreover, understanding the specific contribution given by each dimension, compared to the others, paves the way to set priorities and focused interventions, supporting the organizational strategies and policies for knowledge generation. Indeed, in order to enhance organizational effectiveness, management may propose programs aimed at enhancing the critical processes that, if not supported, could stop the virtuous knowledge generation spiral; implement those processes that need attention despite not being an immediate cause for concern; or even boost those processes on which the organizational identity is grounded (Hatch and Schultz, 2002).

The KMSP-Q can further be used to assess the programs’ effectiveness, by monitoring how these processes evolve over time, or to compare organizations within or across sectors, thus defining benchmarks or specific standard profiles.

### Limitations and Direction for Future Research

The aim of this paper was to integrate the literature regarding the SECI model, by proposing a multidimensional questionnaire within this conceptual framework and examining its structure and construct validity. Although the dimensionality of the KMSP-Q was mainly addressed consistently with Nonaka’s framework, further dimensions could be added and retested for construct validation. For instance, the inter-organizational knowledge exchange could be included (Martin-de-Castro et al., 2008). Additionally, the cross-sectional design precludes the ability to make statements of causal relationships on organizational performance indicators, and future studies utilizing time-lagged or multisource data are recommended. For instance, a longitudinal design would allow to explore how the knowledge creation modes develop and their interaction across time, thus offering empirical grounding to the spiral dynamism the SECI assumes (Nonaka, 1994). For instance, such a design would allow scholars and practitioners to better understand the specific contribution of each dimension in the interplay with the others, also in relation to different outcomes (e.g., learning or performance or innovation at the individual, team, and the organizational level), and what happens in case of change of level (e.g., when the organization invests on one facet, for instance, introducing new practices or through training; or when one of them is particularly weak).

Also, the single-country setting and the specific health sector for Study Two could limit the generalizability of our findings, due to local social practices rooted in contexts (Nonaka and von Krogh, 2009) and the embeddedness of tacit and implicit knowledge (Li and Gao, 2003). Thus, research would benefit from replications in other national or organizational contexts. We further believe that, given the nature of tacit knowledge and difficulty in unveiling it, research would benefit from the adoption of mixed methods.

Overall, this study presents some encouraging results that could stimulate additional inquiry. For instance, modes related to different qualities of knowledge (transformative vs. transfer) do not seem to differently relate to innovation, as some scholars have suggested (Byosiere and Luethge, 2008). Thus, future research should specifically compare innovation outcomes related to both the generation (e.g., creativity) and implementation phases (e.g., new products/services introduced to the market). In addition, multilevel research could study in depth the ontological feature of the model, simultaneously examining the individuals’ and team’s shared perceptions.

## Data Availability Statement

The datasets generated for this study are available on request to the corresponding author.

## Ethics Statement

The studies involving human participants were reviewed and approved by Comitato I.R.B. (Institutional Review Board), Department of Psychology, Sapienza University of Rome (Prot. No. 0000151). The patients/participants provided their written informed consent to participate in this study.

## Author Contributions

MF and BB developed the research project and reviewed the literature. AC carried out the data analysis. GP reviewed the manuscript.

## Conflict of Interest

The authors declare that the research was conducted in the absence of any commercial or financial relationships that could be construed as a potential conflict of interest.
