# Supplementary material for: Managing Knowledge in Organizations: A Nonaka’s SECI Model Operationalization
Source: Front Psychol. 2019 Dec 10;10:2730. doi: 10.3389/fpsyg.2019.02730 (PMC6914727; doi:10.3389/fpsyg.2019.02730)
Supplement: Supplementary file 1 [file Data_Sheet_1.pdf]

APPENDIX
The Knowledge Management SECI Processes Questionnaire

		**In this organization…**
MENT1	1	When employees start a new activity, they are supported by supervisors for a specific period of time.
MENT2	2	When facing problems in their work, employees interact with their colleagues constructively.
MENT3	3	Once hired, employees are trained by more experienced colleagues.
MENT4	4	Newcomers are supported in their job for a specific period of time.
MENT5	5	More experienced colleagues provide less experienced colleagues with constructive feedback about their work.
MENT8	6	There are moments dedicated to the sharing of opinions between colleagues.
SHA2	7	Colleagues are open to share knowledge and skills.
SHA3	8	Each one’s know-how is made available to colleagues to deal with the problems that may arise.
SHA4	9	We share useful knowledge to improve job results.
SHA5	10	Colleagues make their professional experiences available, if someone has difficulty in completing their work.
SHA6	11	The knowledge base needed to carry out one's work is always accessible.
SHA7	12	Colleagues are willing to share their knowledge and skills.
REFL1	13	Individuals are willing and open to changing the way they work in order to improve performance.
REFL2	14	We can learn and acquire benefits from experience.
REFL3	15	It is a good habit to change working methods in light of the results achieved.
REFL4	16	We monitor the effectiveness of our performance in order to plan future actions.
REFL8	17	Reflecting upon past experiences allows us to improve the methodologies in use.
REFL9	18	At the end of each project, we examine the mistakes made in order to prevent their repetition in the future.
MEM2	18	Good practices are collected in databases so that employees may consult them when needed.
MEM3	20	Managers believe that successes, failures and past events should always be considered as examples for future decisions.
MEM4	21	Handbooks are produced for internal use on how to carry out activities.
MEM5	22	We produce a lot of useful and informative documents regarding the results achieved.
MEM7	23	Activities are monitored by collecting and processing relevant data.
MEM8	24	We can consult handbooks or archives to improve our work.
OCOM1	25	Employees are informed about the work of other units.
OCOM2	26	We organize meetings in order to keep us updated.
OCOM4	27	Information is shared between supervisors and employees.
OCOM5	28	We are kept informed about what happens within the organization.
OCOM6	29	Information is shared between the different organizational units.
OCOM8	30	Useful information is effectively disseminated at work.
TEC1	31	The technologies we use promote and support the circulation of ideas.
TEC2	32	Technologies that improve information dissemination and communication between employees are used.
TEC3	33	The use of information technology is guaranteed to all members of the organization.
TEC4	34	We have invested in technologies that facilitate access to information sources

		and databases.
TEC5	35	Technologies allow us to easily share knowledge and information between different units.
TEC7	36	All employees have access to technologies that facilitate the updating of information.
TRAI1	37	Employees' work skills are developed through training.
TRAI2	38	Supervision is a typical tool used to enhance the professional knowledge of employees
TRAI3	39	We are encouraged to take training and professional development courses.
TRAI4	40	Job promotions are supported by training interventions.
TRAI5	41	Training activities are carried out on the basis of employees' needs.
TRAI6	42	New employees are provided with adequate training in order to develop the skills required for their role.
DEV3	43	We have time/resources to reflect upon how to improve our work.
DEV4	44	Employees' extra-work skills are valued.
DEV5	45	We are encouraged to propose new ideas or projects.
DEV6	46	Appropriate resources are available for developing new skills.
DEV8	47	We are supported in tackling the complex challenges of our professional field.
DEV9	48	Talents are valued.

Note. A 5-point Likert-type response format, from 1 (*never or almost never*) to 5 (*very often or always*), is used.
